# Diagnostic and predictive biomarkers of acute rejection after liver transplantation

**DOI:** 10.1097/JS9.0000000000002358

**Published:** 2025-04-03

**Authors:** Qi Pan, Aiwei Zhou, Bingran Wang, Wanglong Xiao, Yunmu Gao, Hongyuan Liu, Jiaqi Song, Yongbo Liu, Yuan Liu, Qiang Xia

**Affiliations:** a Department of Liver Surgery, Renji Hospital, Shanghai Jiao Tong University School of Medicine, Shanghai, China; b Shanghai Engineering Research Center of Transplantation and Immunology, Shanghai, China; c Shanghai Institute of Transplantation, Shanghai, China; d Shanghai Immune Therapy Institute, Shanghai, China

**Keywords:** antibody-mediated rejection, immunosuppression, posttransplantation management, subclinical rejection, T cell-mediated rejection

## Abstract

Liver transplantation serves as a vital therapeutic intervention for individuals suffering from end-stage liver disease globally. A significant complication encountered by liver transplant recipients during the postoperative period is acute rejection, which has traditionally been identified through invasive graft biopsy procedures. Furthermore, assessing the immune status of liver transplant patients is essential for effective posttransplant management and represents a significant advancement toward the personalization of immunosuppressive therapy. Nevertheless, current immunological monitoring after the transplantation predominantly depends on clinical judgment and the measurement of immunosuppressive drug levels, lacking a comprehensive evaluation of actual immune system suppression. In contrast, biomarkers offer a comparatively novel and safer approach for the detection and prediction of transplant rejection, though their clinical application remains constrained due to the absence of prospective validation studies. This review examines the existing literature on potential biomarkers for acute rejection following liver transplantation, and their implications for clinical decision-making.

## Introduction

Since the inception of liver transplantation (LT) in 1963, there has been significant global advancement in LT, establishing it as the primary treatment for end-stage liver diseases[1]. Calcineurin inhibitors (CNIs), including cyclosporine and tacrolimus, significantly reduce the risk of graft rejection and facilitate prolonged graft and patient survival. Additional commonly utilized immunosuppressive agents encompass corticosteroids, basiliximab, mycophenolate mofetil (MMF), and the mammalian target of rapamycin (mTOR) inhibitors such as sirolimus^[^2^]^. However, during the postoperative recovery and follow-up period, a subset of LT recipients could exhibit a high propensity for developing postoperative rejection due to an active immune system^[^3^]^. The likelihood of an LT recipient experiencing acute rejection within the initial 5 years following LT exceeds 50%^[^4^]^. The presence of acute rejection and immunosuppressants is associated with decreased survival of patients and grafts^[^5^]^. A subset of patients, approximately 5%–10%, who develop acute rejection may ultimately progress to liver failure, contributing to nearly 10%–20% of mortality cases after LT^[^6^]^. Hence, the clinical significance of diagnosing and predicting acute rejection is paramount for enhancing postsurgical survival rates^[^7^]^. Timely detection or prevention of rejection is crucial in minimizing the risk of graft injury, necessitating the identification of highly sensitive biomarkers^[^8^]^. A biomarker is a characteristic that can be measured and evaluated objectively to indicate normal or pathogenic biological processes or pharmacological responses to a treatment. An ideal biomarker should be obtained easily with minimal risk to patients and have high sensitivity, specificity, and predictive power^[^9^]^. Therefore, detection of acute rejection at early stages through valid and reliable biomarkers would aid in better management of immunosuppressants and an improved prognosis. This review aims to examine the existing literatures on potential biomarkers for acute rejection or graft acceptance post-LT, and to assess their implications for clinical decision-making.

**HIGHLIGHTS**

- Mechanism of acute rejection after liver transplantation.
- Donor-specific biomarkers, omics data, small molecules, and immune cell biomarkers for the risk of acute rejection after liver transplantation.
- Challenges that still need to be addressed such as the efficacy and positive predictive value in different clinical settings.

### T cell-mediated rejection

Diagnosis of acute rejection typically involves the presence of at least two of the following criteria: portal inflammation, venous endotheliitis, and bile duct injury. Histopathologically, acute rejection after LT can be categorized as T cell-mediated rejection (TCMR) and antibody mediated rejection (AMR)^[^10^]^.

TCMR is characterized by the activation of T cells against graft cells, resulting in graft injury marked by pathological features such as bile duct inflammation, portal inflammation, and/or endotheliitis^[^11^]^. In TCMR, host lymphocytes within the allograft or secondary lymphoid organs recognize allo-antigens originating from the transplanted liver. The primary molecules responsible for TCMR are the major histocompatibility complex (MHC) antigens. MHC class I antigens, which are constitutively expressed by all nucleated cells, present intracellular epitopes to cluster of differentiation 8^+^ (CD8^+^) cytotoxic T cells. Conversely, the expression of MHC class II molecules is more restricted, presenting epitopes derived from extracellular material to CD4^+^ helper T cells. The presentation of allo-antigens by antigen-presenting cells (APCs) constitutes a critical phase in the process of graft rejection. After LT, a substantial number of donor APCs are transferred to the recipient, functioning as passenger leukocytes. Dendritic cells (DCs), B cells, and macrophages are capable of acting as APCs, facilitating allorecognition through various pathways. In the direct pathway, host T cell receptors engage directly with HLA on donor APCs. Conversely, the indirect pathway is initiated when APCs present processed donor peptides, primarily donor HLA, to host T cells. Additionally, the semi-direct pathway involves the transfer of membrane components between host and donor cells, or through extracellular vesicles^[^12^]^. Effective allorecognition necessitates T cell activation subsequent to antigen presentation, receptor communication through the binding of costimulatory receptors with their corresponding ligands on APCs (including CD80, and CD86) and stimulation by cytokines within the microenvironment, leading to T cell proliferation, and subsequent damage to the liver allograft^[^13^]^. Although the specific contributions of these allorecognition pathways to immunological outcomes following LT remain inadequately understood, it is widely acknowledged that the priming of naive T cells initiates the activation of the calcium-dependent phosphatase enzyme calcineurin within the cytoplasm of T cells. This activation subsequently leads to the activation of the nuclear transcription factor of activated T cells, which in turn enhances the expression of interleukin-2 (IL-2). IL-2 serves as a crucial stimulus for T cell proliferation. The prevailing theory is that TCMR results from an immunological imbalance marked by an abundance of T help 1 (Th1) cytokines, including interferon-γ (IFN-γ), tumor necrosis factor-α (TNF-α), and IL-10, which target hepatic cells, causing tissue damage and ultimately graft failure^[^14^]^.

### Antibody mediated rejection

**Figure 1.**
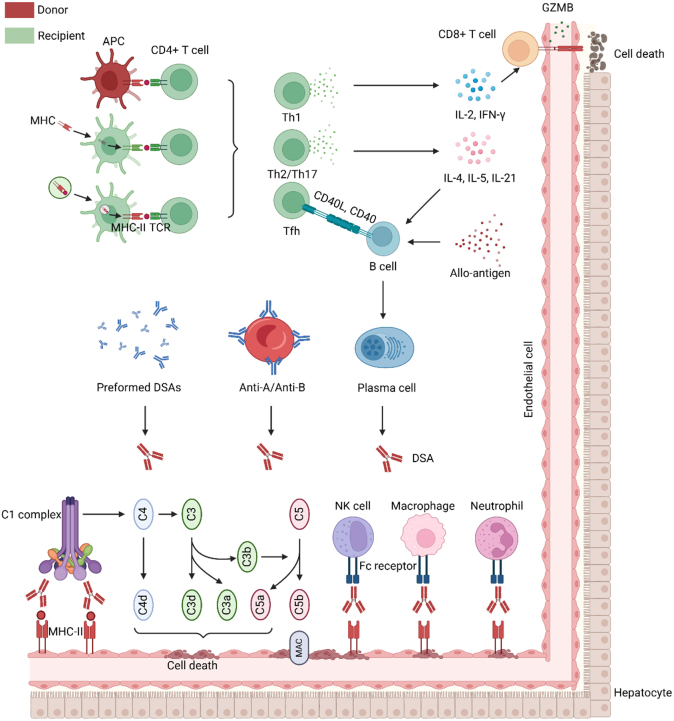
Mechanism of acute rejection after liver transplantation. Following liver transplantation, recipient T and B cells recognize allogeneic antigens. T cell recognition occurs via three distinct pathways. The direct pathway involves the interaction between the recipient TCR and MHC on donor APCs. The indirect pathway entails the internalization of donor MHC antigens by recipient APCs, which subsequently process and present these antigens as peptides to host T cells. The semi-direct pathway involves the interaction between donor MHC and recipient TCR facilitated by vesicle transport. Upon successful allogeneic recognition, CD4^+^ T cells become activated and secrete IL-2 and IFN-γ, which in turn activate CD8^+^ T cells to eliminate target cells. B cells recognizing allogeneic antigens can also be activated through the release of cytokines such as IL-4, IL-5, and IL-21, or via CD40-CD40L interactions, leading to their differentiation into plasma cells. The DSAs synthesized and released by plasma cells, as well as preformed DSA and anti-A/anti-B antibodies, could bind to recipient MHC, initiating the classical complement pathway or recruiting innate immune cells, such as neutrophils, macrophages, and NK cells, to mediate target cell destruction. The classical complement pathway encompasses several key processes: opsonization, where C4d and C3d bind to target cells, thereby marking them for clearance by the innate immune system; the generation of anaphylatoxins, specifically C3a and C5a, which serve as potent chemotactic agents that recruit inflammatory cells and potentially induce tissue damage; and the assembly of the C5b6789 MACs, which compromise cellular integrity by creating pores in the cell membrane. APC, antigen presenting cell; CD, cluster of differentiation; DSA, donor-specific human leukocyte antigen antibody; Fc, fragment crystallizable; GZMB, granzyme B; IFN-γ, interferon-γ; IL, interleukin; MAC, membrane attack complex; MHC, major histocompatibility complex; NK, natural killer; TCR, T cell receptor; Th, T helper.

AMR in LT recipients is a rare occurrence, accounting for less than 5% of cases. Diagnosis of AMR can be confirmed by the presence of donor-specific human leukocyte antigen (HLA) antibodies (DSAs) in serum, characteristic histological findings such as neutrophil and macrophage margination, thrombotic microangiopathy in the microcirculation, and positive complement C4d staining of the endothelium. AMR and TCMR predominantly occur around blood vessels and the biliary tract, rather than within hepatocyte. This is largely due to the specificity of immune cell infiltration, the immunogenicity of blood vessels and the biliary tract, and the liver’s immune surveillance mechanisms. Consequently, the Banff classification can be utilized in clinical settings to assess the degree of vascular injury caused by rejection. The Banff criteria primarily evaluate the extent of inflammation in bile ducts, blood vessels, portal areas, and other regions susceptible to acute rejection. An increased Banff score, indicative of more severe rejection, is associated with a higher degree of inflammatory cell infiltration or degeneration in bile ducts, subendothelial inflammatory cell infiltration, and mixed inflammatory cell infiltration within the portal area^[^15^]^. The pathogenesis of AMR is believed to be primarily mediated by the Fc fragment of DSAs, although experimental data suggest that endothelial cell proliferation and activation may also occur independently of antibody involvement^[^16^]^. The immunological mechanism underlying the development of de novo DSA involves the activation of B cells, the activation of follicular helper T cells, the secretion of antibodies by plasma cells, and other classical antibody production processes. Some studies have indicated that the number of mismatches in HLA functional epitopes at each locus is closely associated with the generation of de novo DSAs. For instance, the number, intensity, and persistence of de novo DSAs generated by HLA-DR β 3/4/5 stimulation are greater than those associated with class I HLA in kidney transplantation^[^17^]^. Recently a retrospective study of 244 pediatric LT recipients also reported that mismatch epitope load could predict rejection and de novo-DSA-free survival^[^18^]^. In contrast to kidney or heart transplantation, where HLA matching is crucial for graft survival, liver allografts have traditionally been regarded as immunologically privileged, exhibiting clinical outcomes that suggest a relative tolerance to HLA mismatches. However, recent advancements in understanding the pathogenesis of de novo DSAs have instigated a paradigm shift. Particular combinations of HLA class II epitope mismatches, especially within the DR/DQ loci, are significantly associated with the development of de novo DSAs, even in patients previously considered to have “acceptable” low-resolution HLA mismatches. Furthermore, in liver transplant recipients with ABO-incompatible blood groups, anti-A or anti-B lectins may bind to the corresponding antigens on the graft’s vascular endothelial cell surfaces, forming immune complexes that subsequently activate the immune system and lead to rejection^[^19^]^. Prior to and following LT in patients with ABO blood group incompatibility, it is essential to assess the titers of anti-A and anti-B antibodies. The administration of rituximab should be considered if deemed necessary. Traditionally, it is posited that antigens either migrate autonomously or are transported by APCs to peripheral lymphoid organs, where they are subsequently recognized by B cells. This recognition induces B cell proliferation, maturation, and differentiation into plasma cells, which serve as the source of DSAs^[^20^]^. The process of antibody-mediated complement fixation plays a crucial role in the pathogenesis of AMR by facilitating the recruitment of inflammatory cells by chemo-attractants within the graft, enhancing the secretion of proinflammatory molecules by endothelial cells, and amplifying immune responses triggered by graft antigens. The resultant graft injury also involves the cytolysis and apoptosis of target cells, phagocytosis of immune complexes via complement receptors on macrophages, vasospasm and edema mediated by the release of prostaglandin E2 or histamine from macrophages, and intravascular thrombosis initiated by endothelial synthesis of procoagulants^[^21^]^. Furthermore, antibodies have the ability to induce target cell lysis through complement-independent mechanisms involving the low-affinity CD16 receptor on natural killer (NK) cells and macrophages^[^21^]^. DSAs are known to activate complement pathways and bind to endothelial antigens, thereby serving as potential biomarkers for AMR. Nonetheless, the correlation between DSA presence and AMR, as well as complement C4d positivity, is not absolute^[^22^]^. This underscores the necessity of employing combined diagnostic criteria and highlights the continued importance of biopsy in diagnosing AMR, despite advancements in serologic testing (Fig. 1).

### Clinical values of potential biomarkers in acute rejection

The significant impact of acute rejection necessitates the timely identification or anticipation of rejection following LT. Acute rejection typically manifests between 5 and 30 days post-surgery, with a peak incidence occurring between 7 and 10 days postoperatively. The likelihood of acute rejection is heightened in cases of inadequate immunosuppression or poor patient adherence to prescribed regimens. Clinically, acute rejection presents with nonspecific symptoms, including fever, fatigue, and anorexia. Laboratory findings often reveal abnormal liver function, characterized by elevated transaminases (ALT/AST), typically ranging from two to five times the upper limit of normal, increased bilirubin levels, and elevated γ-glutamyl transferase and alkaline phosphatase. Despite these indicators, specific diagnostic signs are lacking, necessitating the exclusion of alternative etiologies. In the presence of these symptoms, normal hemodynamic parameters, non-elevated infection markers, and the exclusion of biliary complications, clinicians should consider further diagnostic evaluations to ascertain the presence of acute rejection^[^23^]^. In addition to acute rejection, a subset of LT recipients may be prone to subclinical rejection (SCR)^[^24^]^. SCR is a prevalent form of occult graft injury following LT, characterized by histological evidence of rejection despite normal liver function, detectable only through protocol liver biopsy. A recent study revealed an 18.6% incidence of SCR in LT recipients undergoing protocol biopsy, along with a heightened prevalence of allograft fibrosis^[^25^]^. Recipients with SCR often exhibit no apparent clinical symptoms and have good graft function. Liver enzymes such as alanine aminotransferase (ALT) and aspartate aminotransferase (AST) alone are insufficient for providing a definitive diagnosis, typically rising only after rejection and liver injury have already taken place^[^26^]^. Therefore, protocol liver biopsy is considered the preferred method for diagnosing SCR. However, liver biopsy is associated with potential complications and is not commonly utilized for routine monitoring^[^27^]^. As a result, there is a need for the development of efficient, reproducible, readily accessible, and less invasive biomarkers to detect and predict the progression of acute rejection. Monitoring SCR changes through biomarkers could inform the judicious administration of immunosuppressive medications. LT recipients often face the challenge of lifelong immunosuppressant therapy, which can result in significant healthcare and clinical expenses unless discontinuation is required because of malignancy, severe infection, or other life-threatening complications^[^28^]^. A study in South Korea included 1469 recipients undergoing LT and founded 21.2% of them experienced tumor recurrence, who were administered immunosuppressants after the surgery^[^29^]^. Another study in China revealed that 69.1% and 47.5% of the LT recipients had experienced Epstein–Barr virus and cytomegalovirus infections with the use of immunosuppressants, respectively^[^30^]^. Cardiovascular disease is prevalent among a substantial proportion of recipients, largely due to the exacerbation of hypertension, diabetes, and hyperlipidemia resulting from immunosuppressant treatment. CNIs play a substantial role in the development of advanced chronic kidney disease in a significant portion of long-term LT recipients, potentially necessitating renal replacement therapy and kidney transplantation^[^31^]^. Improved patient outcomes may be achieved through the reduction or cessation of immunosuppressant exposure. It has been recognized that certain individuals may thrive without immunosuppression, either due to nonadherence or the discontinuation of immunosuppressive therapy for the management of infections or lymphoproliferative disorders^[^32^]^. The concept of “immune tolerance” refers to the state of a patient exhibiting normal graft function in the absence of immunosuppressive medication^[^33^]^. The induction of immune tolerance following LT can render the recipient’s immune system permanently unresponsive to donor liver-specific antigens while maintaining normal immune responses to other pathogens. Achieving successful immune tolerance can potentially allow for the reduction or cessation of immunosuppressive therapy, thereby mitigating the adverse effects associated with long-term medication use, such as increased susceptibility to infections, metabolic disturbances, and heightened tumor risk. Strategies to induce immune tolerance encompass several approaches, including the infusion of regulatory T cells, leveraging the paracrine effects of mesenchymal stem cells to inhibit DC maturation, and employing costimulatory blockers or mTOR inhibitors. Presently, the predominant clinical approach involves the “gradual withdrawal” of immunosuppressants, wherein the dosage of tacrolimus or cyclosporine is progressively reduced following surgery. During withdrawal of the immunosuppressants for establishment of immune tolerance, the recipients may suffer from rejection, which can be predicted by distinctive biomarkers^[^34^]^. The identification of reliable biomarkers for identifying individuals likely to exhibit a tolerogenic response is crucial for effectively inducing immune tolerance^[^35^]^.

Numerous biomarkers have been investigated to identify specific indicators of graft rejection following LT. However, the utility of these biomarkers has been constrained by significant overlap with other conditions, and only a limited number have been validated to date.

## Biomarkers for diagnosis and prediction of acute rejection in LT recipients

**Figure 2.**
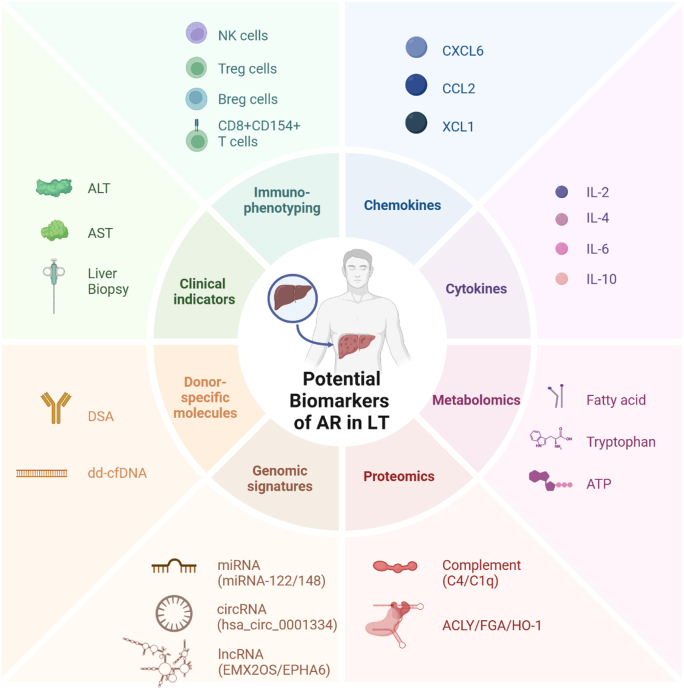
Potential biomarkers of acute rejection in liver transplantation. The potential biomarkers associated with acute rejection in liver transplantation are categorized into several areas. ACLY, ATP citrate lyase; ALT, alanine aminotransferase; AST, aspartate aminotransferase; ATP, adenosine triphosphate; AUC, area under the curve; Breg, Regulatory B cell; CCL, C-C motif ligand; CD, cluster of differentiation; circRNA, circular RNA; CXCL, C-X-C motif ligand; dd-cfDNA, donor-derived cell-free DNA; DSA, donor-specific human leukocyte antigen antibody; EMX2OS, empty spiracles homeobox 2 opposite strand; EPHA6, erythropoietin-producing hepatocellular kinase receptor A6; FGA, fibrinogen alpha chain; HO-1, heme oxygenase-1; IL, interleukin; lncRNA, long non-coding RNA; LT, liver transplantation; miRNA, microRNA; Treg, regulatory T cell; XCL, X-C motif ligand.

Currently, a variety of molecular and cellular biomarkers with high sensitivity and specificity related to the pathophysiology of acute rejection are being utilized to assess the immune status of LT recipients and inform the optimization of immunosuppressant dosages. They can be broadly classified into four categories: donor-specific biomarkers, omics data, small molecules (cytokines and chemokines), and immune cell biomarkers, as further elaborated upon in the subsequent discussion^[^36^]^ (Fig. 2, Table1).

**Table 1.** Representative biomarkers in predicting acute rejection after liver transplantation

Type	Biomarker	PPV	NPV	Reference
Donor-specific biomarkers	dd-cfDNA	66.7%	87.8%	39
	DSAs	NA	NA	49
Multi-omics data	36 gene panel	47%	87%	53
	59 gene panel	54%	89%	54
	3 miRNA panel	75%	93%	62
	470 proteoform panel	NA	NA	67
	C4, C1q	74%	94%	69
	ATP	NA	NA	74
Cytokines and chemokines	IL-6	NA	NA	78
		
	CCL2	NA	NA	82
Immunophenotyping	KIR^+^ NK cells	NA	NA	90
	CD4^+^CD25^high^ Tregs	58%	95%	92
	CD154^+^ T cells	64%	92%	95
	GZMB^+^CD19^+^ B cells	NA	NA	101

PPV and NPV are listed when applicable.

ATP, adenosine triphosphate; C, complement; CCL, C-C motif ligand; CD, cluster of differentiation; dd-cfDNA, donor-derived cell-free DNA; DSA, donor-specific human leukocyte antigen antibody; GZMB, granzyme B; IL, interleukin; KIR, killer cell immunoglobulin-like receptor; LT, liver transplantation; miRNA, microRNA; NA, not available; NK, natural killer; NPV, negative predictive value; PPV, positive predictive value; Treg, regulatory T cell.

### Donor-specific biomarkers

Circulating free DNA originating from donor cells, also known as donor-derived cell-free DNA (dd-cfDNA), is constantly shed from the allograft itself into blood circulation after LT^[^37^]^. Both dd-cfDNA and DSAs have been employed as non-invasive biomarkers, specifically referred to as donor-specific biomarkers, for acute rejection in liver, kidney, and lung transplantation since 1998^[^38^]^. The technology for detecting dd-cfDNA in recipient cell-free DNA, such as quantitative real-time polymerase chain reaction, holds promise as a sensitive tool for identifying dysfunction in transplanted organs. The underlying principle of this assay is to quantify the levels of dd-cfDNA, based on the premise that an injured graft releases elevated levels of dd-cfDNA^[^39^]^. Furthermore, dd-cfDNA is anticipated to reflect current physiological events due to its brief half-life, which ranges from minutes to hours in the circulatory system^[^22^]^. The study conducted by Levitsky *et al*^[^40^]^ aimed to assess the diagnostic potential of dd-cfDNA in LT recipients from both single and multicenter cohorts as a marker for graft integrity and injury progression and predictor of causes of graft dysfunction. Phenotypes of treated biopsy-proven acute rejection (N = 57), normal function (N = 94), and acute dysfunction without rejection (N = 68) were stratified into training and test sets. In the training set, the levels of dd-cfDNA showed significant differences between acute rejection versus normal function (area under the curve [AUC] 0.95, cutoff 5.3%) and acute rejection versus acute dysfunction without rejection (AUC 0.71, cutoff 20.4%). Applying these cutoff values to the test set yielded an accuracy of 87% and a negative predictive value (NPV) of 100% for distinguishing acute rejection from normal function, and an accuracy of 66.7% and NPV of 87.8% for distinguishing acute rejection from acute dysfunction without rejection^[^40^]^. Serial blood samples collected from LT recipients showed gradual increases in dd-cfDNA levels prior to the development of graft dysfunction and decreases after rejection treatment^[^41^]^. Therefore, the progressive elevation of dd-cfDNA may serve as an indicator of preclinical graft injury in the absence of abnormal liver function tests, with the highest levels observed during rejection episodes. The quantitative fluorescent polymerase chain reaction identified a significantly higher number of short tandem repeats in individuals who later experienced acute rejection compared to non-rejectors, even as early as 1–2 weeks prior to rejection. This trend was also observed in the rapid evaluation of deletion or insertion polymorphisms, as supported by a study conducted by Zhao *et al*^[^41^]^ analyzing the percentage of dd-cfDNA to total cell-free DNA in 76 LT recipients. A substantial portion of current acute rejection cases could be accurately detected with over 90% sensitivity and specificity using dd-cfDNA. However, the ability of dd-cfDNA to differentiate acute rejection from graft dysfunction caused by other factors may decrease to 75% sensitivity and 60% specificity. In summary, the inherent characteristics of dd-cfDNA may provide a more accurate and earlier indication of rejection activity compared to traditional liver enzymes. The primary advantage of dd-cfDNA over tissue biopsy lies in its noninvasive nature, ease of repetition, and high NPV, which collectively facilitate the avoidance of unnecessary diagnostic procedures and biopsies. Owing to its advanced technical maturity and substantial clinical evidence, it is poised to assume a leading role in the realization of commercial testing. A 2022 study demonstrated that continuous monitoring of blood samples can detect an increase in dd-cfDNA prior to graft dysfunction, indicating its feasibility for dynamic monitoring immediately after LT^[^38^]^. Future prospective multicenter trials are anticipated to elucidate the optimal application of dd-cfDNA in the detection and prediction of graft dysfunction.

DSAs have been established as a significant factor in kidney, heart, liver, and lung transplant rejection, serving as a diagnostic indicator for antibody-induced acute rejection^[^42,43^]^. Notably, in most LT programs, the liver is transplanted without prior cross-match results, predicated on the assumption that the liver exhibits a degree of tolerance to the humoral immune response^[^44^]^. A recent study indicated that a positive cross-match may elevate the risk of early graft loss in LT recipients, potentially induced by circulating DSAs^[^45^]^. DSAs can target either class I or class II HLA or non-HLA antigens, such as the angiotensin II type-1 receptor^[^46^]^. Class II DSAs may influence clinical outcomes due to the upregulation of class II antigen expression on the portal microvasculature, centrilobular endothelium, bile ducts, and hepatocytes, which occurs following an initial insult to the allograft^[^47^]^. These DSAs can be pre-formed prior to LT or develop posttransplantation, a phenomenon referred to as *de novo* DSAs. It is imperative to recognize that medication non-adherence and suboptimal immunosuppression levels are significant risk factors for the development of *de novo* DSAs^[^48^]^. Recent research has demonstrated a correlation between pretransplant DSAs and various adverse outcomes, including allograft portal fibrosis, an elevated risk of biliary strictures, AMR, TCMR, and allograft loss following LT^[^49^]^. A recent meta-analysis, which included studies on 2016 LT recipients with *de novo* DSAs, indicated a heightened risk of complications such as graft loss and chronic rejection (odds ratio [OR] 3.61), as well as allograft rejection alone (OR 6.43)^[^44^]^. These complications were characterized by subtle portal inflammation and distinctive patterns of perivenular fibrosis, despite the presence of normal or near-normal liver function tests, in comparison to recipients without de novo DSAs^[^50^]^. Several single-center retrospective case reports have evaluated the efficacy of AMR therapy in preventing graft rejection. Consequently, the presence of DSAs may serve as a biomarker for identifying patients who should avoid immunosuppression weaning post-LT. Screening for DSAs prior to LT may assist in identifying patients who will necessitate close monitoring of DSA levels. However, in the absence of large cohort studies, there are no specific recommendations, leaving the decision to screen for DSAs before LT to the discretion of individual LT programs. It is advised that DSAs should be assessed in cases where patients exhibit severe TCMR, steroid-resistant TCMR, or allograft dysfunction of unknown etiology, due to the potential presence of concomitant AMR.

### Multi-omics data

Peripheral blood samples can be utilized to detect genomic signatures indicative of allograft acute rejection through the analysis of gene expression profiles. Elevated levels of various types of DNA and RNA, including microRNA, long non-coding RNA (lncRNA), and circular RNA (circRNA), in LT recipients have been correlated with episodes of acute rejection^[^51^]^. This correlation facilitates the development of specific genomic panels for the accurate detection of acute rejection^[^52^]^. Levitsky *et al*^[^53^]^ utilized blood gene profiling to identify a panel of 36 genes capable of detecting messenger RNA (mRNA), which distinguishes patients with biopsy-confirmed rejection from those with normal liver function up to three months prior to the biopsy. This panel demonstrated positive and NPVs of 47% and 87%, respectively. Additionally, the researchers expanded the panel to include 59 genes, successfully differentiating acute rejection patients from those with other causes of liver injury^[^54^]^. However, the study lacked surveillance biopsies with normal histology that could be paired with blood samples to more accurately define stable graft function^[^55^]^. To elucidate network modules implicated in rejection, Ningappa *et al*^[^56^]^ employed integrative machine learning techniques to amalgamate transcriptomics data with a high-quality reference human protein interactome. Consequently, the authors have demonstrated a proof of concept for a methodology that can concurrently identify blood-based module biomarkers of LT outcomes at various time points and target corresponding druggable mechanisms, furnishing insights into dysregulated molecular profiles at an individual level^[^56^]^. Several of these mechanisms can be relatively easily implemented by adjusting existing immunosuppression strategies. Recently Urie *et al*^[^57^]^ reported on a scaffold implant serving as detector of acute rejection in heart and skin transplantation, which contributed to the identification of 18 differentially expressed genes between pre-injury acute rejection and late acute rejection. Hence, the device may help provide an insight into remote evaluation of early risk of acute rejection and reduce the frequency of routine biopsy^[^57^]^.

Regarding RNA biomarkers, numerous studies have demonstrated that specific RNA species are associated with the progression of acute rejection, either directly or indirectly, and can serve as indicators even before clinical rejection occurs^[^58^]^. Certain microRNAs, which are small non-coding RNA sequences involved in the regulation of transcription processes, have been linked to acute liver rejection through the upregulation of the pro-inflammatory transforming growth factor-β pathway and the downregulation of the forkhead box protein P3 pathway in regulatory T cells (Tregs)^[^59^]^. Over the past decade, a novel mechanism of intercellular communication mediated by extracellular vesicles has been identified^[^60^]^. These membrane-bound vesicles, ranging from 0.05 to 1 μm in size, play a role in normal physiological processes and are also implicated in various pathological conditions. Cells release these entities into the bloodstream and other bodily fluids, facilitating the transport of bioactive molecules from their origin to recipient cells through both phagocytic and non-phagocytic pathways^[^61^]^. This process enables the direct delivery of their contents into the cytoplasm of target cells. Farid *et al*^[^60^]^ analyzed serum and liver samples from 107 patients and identified two microRNAs (miRNA-122 and miRNA-148) derived from hepatic cells as early serological biomarkers. Additionally, Millán *et al*^[^62^]^ found that the levels of three microRNAs (miRNA-181a-5p, miRNA-155-5p, and miRNA-122-5p) are positively correlated with the severity of rejection. However, individual RNA expression levels are highly susceptible to dietary, pharmacological, and immunological influences, as well as human factors such as the timing of sample collection^[^63^]^. This necessitates the integration of multiple RNA markers to more accurately interpret patient status. Shaked *et al* have developed a 31-panel microRNA test using whole blood samples from patients post-LT, which enhances the accuracy of diagnosing and predicting acute rejection^[^64^]^. Similar to dd-cfDNA, miRNAs are among the most extensively studied biomarkers currently. The cost of detecting miRNAs has been decreasing due to advancements in high-throughput sequencing technologies, facilitating the availability of commercial detection kits. Nonetheless, the efficacy, sensitivity, and specificity of RNA biomarkers remain subjects of debate, primarily due to the limited scale of clinical trials and the paucity of studies in this area. Accordingly, more studies are urgently required to elucidate the roles of RNAs in rejection and search for predictors of acute rejection^[^65^]^.

Given the relatively low sensitivity and specificity of single-factor models, recent research has increasingly focused on additional influencing factors involved in the progression of rejection, particularly in the fields of proteomics and metabolomics^[^66^]^. In addition to antibodies, cytokines, and chemokines, Li *et al*^[^67^]^ observed an upregulation of 470 proteins and a downregulation of 50 proteins in mouse LT models, primarily associated with leukocyte migration and fatty acid metabolism, respectively. However, murine models frequently do not accurately represent human hepatic pathology, and the sample sizes in these studies are limited, necessitating further research involving larger cohorts of human subjects^[^68^]^. Melani *et al*^[^69^]^ have developed a 24-protein panel derived from the peripheral blood of patients, whereas Massoud *et al*[70^]^ have identified complement C4 and C1q, integral to the innate immune system, in the serum of LT recipients as potential diagnostic markers for acute rejection post-LT. The innovative approach of direct proteoform measurement, as opposed to less specific epitope- or peptide-based methods, holds promise for advancing patient care^[^71^]^. This method enables the early detection of specific indicators of immune activation versus immune quiescence, thereby facilitating the personalization of immunosuppressive therapy monitoring and modulation for LT recipients^[^72^]^.

Metabolomics endeavors to identify biomarkers linked to altered metabolic pathways in biological fluids and tissues, thereby enhancing our understanding of the downstream effects of genes and proteins. This approach demonstrates significant diagnostic potential due to the involvement of various metabolites in acute rejection, as revealed by numerous gene ontology and Kyoto Encyclopedia of Genes and Genomes analyses. In their study, Frediani *et al*^[^73^]^ included children with (n = 18) and without (n = 25) acute cellular rejection^[^73^]^. Linear regression analysis identified 510 metabolites that significantly differentiated between the groups. Pathway analysis indicated that bile acid biosynthesis and tryptophan metabolism were the top two differentiating pathways. Additionally, network analysis highlighted tryptophan, which was clustered with liver enzymes and steroid use. Ultimately, 11 metabolites associated with tryptophan metabolism were identified, including cysteine, which is implicated in the modulation and activation of T cells, as well as nine metabolites involved in bile acid synthesis. However, the researchers did not provide a comprehensive elucidation of all the metabolites. Schulz-Juergensen *et al*^[^74^]^ reported significantly elevated levels of intracellular adenosine triphosphate in four recipients experiencing rejection compared to non-rejectors, although a definitive cut-off value could not be established due to substantial interindividual variability. Nonetheless, a notable limitation of metabolomics research is the relatively small sample sizes typically employed. Furthermore, there is a paucity of studies specifically addressing LT recipients, which complicates the ability to make generalizable extrapolations for this demographic, given the heterogeneity among different populations. In conclusion, proteomics and metabolomics data are not directly applicable for AR diagnosis because of their low sensitivity and specificity, which must be integrated with other markers for effective use.

### Cytokines and chemokines

Given that the implant initially triggers an inflammatory response in acute rejection, the levels of inflammation-related small molecules (cytokines and chemokines) are expected to rise following rejection, even prior to the manifestation of symptoms^[^75^]^. Studies have indicated that recipients with acute rejection following LT exhibit elevated circulating levels of soluble IL-2 receptors compared to transplant recipients without rejection. This is attributable to the fact that IL-2 expression levels partially reflect the degree of T cell activation^[^76^]^. Similarly, cytokines such as IL-6, IL-10, and IL-17, secreted by various T cell subsets, follow this trend^[^77^]^. However, despite IL-6 being a commonly utilized clinical marker, its strong correlation with C-reactive protein, which indicates nonspecific inflammation, undermines its reliability as a biomarker when compared to other cytokines^[^78^]^. The concentration of IL-6 in LT recipients can be significantly influenced by the onset of viral or bacterial infections, as well as by the administration of antibiotics and immunosuppressive agents such as high-dose steroids and cyclosporin A^[^79^]^. Conversely, other cytokines have shown considerable potential through the rapid and comprehensive investigation of the mechanisms underlying acute rejection. Multiple studies assessing the levels of T-helper cell cytokines, such as IL-4, IL-10, IL-15, IFN-γ, and TNF-α, at various time points before and after LT have indicated that the levels of both Th1 and Th2 cytokines may increase shortly after LT, thereby reflecting the inflammatory status of the recipients^[^80^]^. Interestingly, Th2 cytokines, such as IL-4, exhibit significant predominance in non-rejectors. Conversely, in cases of biopsy-proven acute rejection, the levels of Th1 cytokines tend to be marginally elevated compared to Th2 cytokines, potentially aiding in the identification of recipients with a higher propensity for rejection. Furthermore, immunohistochemical analysis targeting transcription factors that characterize T cell populations indicates a polarization toward Th1 lymphocytes in acute rejection^[^81^]^. However, interpreting circulating cytokine concentrations poses significant challenges due to their unique half-lives. Consequently, it is advantageous to further characterize the cytokine immune response by detecting and analyzing the expression of cytokine precursors.

Specifically, small-sized chemokines, a distinct category of cytokines, play a crucial role in facilitating the migration and positioning of immune cells in LT recipients. Among the more than 40 chemokines identified to date, a significant proportion can be categorized as inflammatory or inducible chemokines, which are expressed in transplants in response to antigens or proinflammatory cytokines. Examples of these chemokines include C-X-C motif ligand 6, C-C motif ligand 2 (CCL2), and X-C motif ligand 1 (XCL1). Conversely, a smaller subset of chemokines involved in immune surveillance can be classified as homeostatic or constitutive. These chemokines, such as CXCL12 and CCL21, play crucial roles in the trafficking of DCs and the regulation of lymphocytes^[^76,82^]^. Despite the evident differences between rejection and non-rejection samples, neither individual circulating cytokine levels nor a combination of these levels demonstrated adequate predictive value to be deemed a reliable diagnostic test in the context of clinically suspected acute rejection^[^83^]^. For patients exhibiting signs of hepatic dysfunction and inflammation, it is also not advisable to use cytokines or chemokines as biomarkers to distinguish between rejectors and non-rejectors^[^84^]^.

In summary, pro-inflammatory and immunoregulatory cytokines have been extensively investigated as biomarkers for predicting acute rejection. Although the majority of these cytokines exhibit elevated expression levels during acute rejection, their ability to distinguish between acute rejection and infections remains limited, thereby constraining their clinical utility. Furthermore, many inflammatory factors have not been validated through large-scale multicenter studies, and there is a lack of standardized detection protocols, which hinders their clinical application and the development of diagnostic kits. Additional research is required to ascertain whether early cytokine panel analysis possesses adequate precision to predict subsequent rejection, thereby justifying a preemptive escalation in immunosuppressive therapy for selected patients.

### Immunophenotyping

The assessment of specific T cell and B cell immunophenotypes, based on the CD markers on the surface or within immune cells, has long been a focal point of research and has been implemented in clinical practice, particularly in the context of transplant rejection^[^85^]^.

Although intrahepatic innate immune cells are crucial in preventing rejection following LT, clinical cases leveraging their functions to reduce immunosuppressive agent dosages are rarely reported. Typically, intrahepatic innate immune cells exist in a balanced state between pro-inflammatory and anti-inflammatory activities. Under normal, resting conditions, these cells primarily induce intrahepatic immune tolerance. Conversely, under inflammatory conditions, they promote immune responses and release inflammatory cytokines, potentially leading to acute rejection. Further investigation into the critical factors influencing the transformation of intrahepatic innate immune cells between these two states is crucial for elucidating the mechanisms underlying acute rejection^[^86^]^. For instance, studies utilizing animal models of transplantation have demonstrated the involvement of NK cells in the process of transplant rejection. Mice that are deficient in both T and B cells retain the capacity to reject allogeneic cells^[^87^]^. Furthermore, recombination-activating gene (RAG) knock-out mice, when further primed with IL-15, are able to reject skin allografts^[^88^]^. NK cells appear to significantly influence the alloantigen-specific T cell response, as recipient NK cells serve as an early source of IFN-γ during transplant rejection, thereby promoting allogeneic T cell responses. Early investigations into HLA-C disparity between donor and recipient demonstrated a correlation between HLA-C compatibility, a reduction in acute rejection rates, and enhanced graft survival^[^89^]^. Furthermore, elevated expression of KIR (killer cell immunoglobulin-like receptor) receptors on recipient NK cells, which are responsible for recognizing HLA-C epitopes, was also associated with improved graft survival and fewer rejection episodes^[^90^]^. However, research on KIR-HLA class I mismatches has not conclusively established a correlation with graft rejection or survival.

CD4^+^CD25^high^ Tregs are recognized for their role in establishing tolerance post-LT and serve as a well-established marker of rejection^[^91^]^. Li *et al*^[^92^]^ found that the number of CD4^+^CD25^high^ Tregs was significantly higher in the peripheral blood and the portal areas of grafts in tolerant patients but lower in recipients with acute rejection. Additionally, activated proliferating lymphocytes are known to express CD25 and CD71 on their surface, with CD69 expression occurring at certain stages^[^93^]^. However, lymphocyte activation markers can be upregulated not only by inflammation and rejection but also by viral and bacterial infections, complicating the determination of the precise cause of elevated activated immune cells^[^94^]^. Ashokkumar *et al*^[^95^]^ have elucidated that the elevation of CD4^+^CD154^+^ T cells and the reduction of CD8^+^CD154^+^ T cells are associated with increased risks of rejection in 58 identically immunosuppressed children who underwent LT. Additionally, they quantified CD154^+^ T cytotoxic memory (Tcm) cells in cryopreserved samples from 214 children under 21 years of age^[^96^]^. Recipient CD154^+^ Tcm cells, induced by stimulation with donor cells, were quantified as a fraction of those induced by HLA-non-identical cells in parallel cultures. The predictive performance metrics included a sensitivity of 84%, specificity of 80%, positive predictive value of 64%, and NPV of 92%, with an AUC of 0.792^[^85^]^. Furthermore, the rapid advancement of single-cell sequencing technologies has led to the identification of more specific cell populations beyond the traditional T cell subset^[^97^]^. A correlation has been identified between CD4^+^CD7^+^ and CD8^+^CD38^+^ T cells and early acute rejection. Additionally, an increase in the proportion of CCR6^+^CD4^+^ T cells, myeloid-derived suppressor cells, and folate receptor gamma 3^+^ Kupffer cells has been observed in rejectors, concomitant with a decrease in CD163^+^ Kupffer cells, apolipoprotein E^+^ Kupffer cells, and granzyme A^+^ Kupffer cells.

Regulatory B cells (Bregs) play a critical role in modulating the immune response across various disease contexts, including transplantation. Although they lack a definitive phenotypic marker or transcription factor, their importance in transplantation is highlighted by their capacity to extend experimental allograft survival, their potential application as tools for immune monitoring, and the promising possibility of their use in cell-based therapies. Furthermore, the ratio of B cell-derived IL-10 to TNF-α has been demonstrated to be predictive of immunological reactivity and acute rejection in kidney and LT^[^98^]^. The assessment of the Breg/B effector balance using this ratio may facilitate the identification of patients who require enhanced immunosuppression and offer mechanistic insights into potential therapeutic interventions^[^99^]^. Granzyme B (GZMB)-producing B cells have been identified as a significant regulatory B cell subset implicated in the pathogenesis of acute rejection. LT recipients experiencing acute rejection exhibited elevated expression of GZMB in CD19^+^ B cells compared to controls, although the increase did not achieve statistical significance^[^100^]^. Consequently, the elevated expression of GZMB in CD19^+^ B cells from liver transplant recipients experiencing acute rejection may serve as a feedback mechanism for the activation of CD4^+^CD25^−^ T cells^[^101^]^.

Overall, these immunophenotyping studies demonstrate high specificity, typically exceeding 90%; however, their sensitivity is relatively low compared to other biomarkers mentioned. Additionally, most immune cell populations are susceptible to variations caused by other diseases, limiting their widespread clinical use. This necessitates further data mining and comparison with existing databases.

## Perspective and conclusion

The aforementioned studies represent a limited review of the described biomarkers in LT. However, there is a deficiency in studies evaluating predictors for rejection, and prospective validation of identified markers is necessary. Biomarkers have often been identified and validated solely through for-cause biopsies. Consequently, the efficacy of these markers in screening all LT recipients remain uncertain, and it is ambiguous whether these markers are biologically relevant or merely indicative of ongoing liver injury^[^102^]^. Another significant challenge is that many identified markers for allograft rejection possess a high NPV, which makes them useful for identifying a state of immune quiescence and facilitating the minimization of immunosuppressive therapy. However, the positive predictive value in numerous instances remains relatively low, necessitating liver biopsy to confirm the presence of ongoing injury, thereby limiting their clinical utility as diagnostic markers. Furthermore, research is required to evaluate the use of multiple biomarkers that assess ongoing rejection at genetic, transcriptional, or immunological levels. While readouts from a single serological test can be beneficial, the application of a multidimensional assay combined with dynamic observation for the prediction and diagnosis of rejection may offer greater specificity in identifying subclinical cellular rejection. By elucidating the alterations in intra-graft molecular pathways during acute rejection, it is hypothesized that a judicious combination of biomarkers will enhance decision-making accuracy and diminish the necessity for invasive liver biopsies in LT recipients. It is increasingly apparent that a single biomarker is insufficient to capture the comprehensive alterations in the immune system associated with organ transplantation. Consequently, a panel of diverse biomarkers is required to accurately assess immunological suppression and to tailor immunosuppressive treatment to the specific needs of patients. Once identified, this panel of biomarkers should undergo validation through extensive multicenter studies to establish its clinical utility.

## Data Availability

Nothing to declare.

## Research registration unique identifying number (UIN)

Nothing to declare.
